# Priorisierung von Geimpften?

**DOI:** 10.1007/s00481-022-00716-8

**Published:** 2022-08-09

**Authors:** Tatjana Hörnle

**Affiliations:** Humboldt-Universität zu Berlin und Max-Planck-Institut zur Erforschung von Kriminalität, Sicherheit und Recht, Günterstalstr. 73, 79100 Freiburg, Deutschland

**Keywords:** Priorisierung in der Intensivmedizin, Impfstatus, Covid-19-Pandemie, Richtlinien für Triage, Triage, Vaccination status, COVID-19 pandemic, Guidelines for resource allocation

## Abstract

Der Beitrag befasst sich mit der Frage, ob der Impfstatus ein Auswahlkriterium sein könnte oder sogar sollte, falls in einer Pandemie bei akuter Ressourcenknappheit Priorisierung unabwendbar wird. Er ordnet und bespricht unterschiedliche Thesen, die dazu vertreten werden. Erstens wird angenommen, dass eine Beschäftigung mit diesem schwierigen Problem nicht erforderlich sei. Zweitens könnte man darauf abstellen, dass heikle Themen besser nicht öffentlich erörtert werden sollten. Nach der dritten, häufig vertretenen Argumentationslinie dürfe auf keinen Fall ein Unterschied zwischen geimpften und ungeimpften Patienten gemacht werden, weil dies mit Menschenrechten kollidiere oder mit dem allgemeinen Grundsatz nicht zu vereinbaren sei, dass Vorverhalten von Patienten in der Intensivmedizin unbeachtlich ist. Viertens empfehlen u. a. Verhaltensökonomen eine Berücksichtigung des Impfstatus zur Verbesserung von Impfquoten. Fünftens ist aus ethischer Sicht zu erwägen, ob eine vorangegangene Impfung aus Gründen der Gerechtigkeit Bedeutung habe müsse. Die Autorin vertritt die sechste These, nämlich dass bei vergleichbarer klinischer Erfolgsaussicht ggf. das Unterlassen einer möglichen und empfohlenen Impfung mangels besserer Auswahlkriterien herangezogen werden dürfe. Sie spricht sich insbesondere gegen die Alternative einer Zufallsentscheidung aus.

## Abstract (en)

**Definition of the problem:**

The article asks whether vaccination status could become relevant if it is unavoidable to prioritize between patients in intensive care units during a pandemic. The aim is to analyze different approaches and arguments in favor of and against the inclusion of vaccination status.

**Arguments:**

The following arguments are assessed: First, it has been argued that it is unnecessary to open this discussion. Second, one could make the point that public debates about touchy subjects should be avoided. A third, frequently expressed opinion claims that physicians must never discriminate between vaccinated and unvaccinated patients, either because this is in conflict with human rights or because this is incompatible with the general principle that patients’ prior conduct does not matter. Fourth, behavioral economists argue that intensive care medicine should take vaccination status into account with the goal to improve the overall numbers of vaccinations. A fifth line of thinking argues that it is more just to take vaccination into account.

**Conclusion:**

The author concludes that the omission to get a necessary and recommended vaccination may be taken into account if patients’ prospects to survive are similar. She points out that lotteries would be worse.

## Einleitung

Die folgenden Überlegungen gelten den Auswahlkriterien, die benötigt würden, wenn eine Pandemie zu nicht behebbarer Ressourcenknappheit auf Intensivstationen geführt hat und priorisiert werden muss, weil die Behandlungsmöglichkeiten nicht mehr ausreichen, um alle Patienten in lebensbedrohlichem Zustand (infolge der pandemischen Erkrankung, anderer Krankheiten oder Verletzungen) zu versorgen. Ich konzentriere mich dabei nur auf *eine* Spezialfrage, nämlich die mögliche Berücksichtigung des Impfstatus als ein zusätzliches Kriterium, das herangezogen werden könnte, sobald eine Impfung entwickelt, zugelassen und in der Pandemie als dringende Handlungsempfehlung beworben wurde. Anlass für die Befassung mit dieser Frage war natürlich die COVID-19-Pandemie (in der Zeitspanne der Jahre 2021 und 2022, seitdem Impfungen verfügbar sind, aber wegen schnell steigender Infektionszahlen eine Überlastung der Intensivstationen befürchtet wurde). Unabhängig von der historischen Einbettung lohnt es sich, das Thema nicht zu den Akten zu legen, denn es ist nicht auszuschließen, dass neue Virusvarianten oder andere Krankheitserreger neue Pandemiewellen oder Pandemien verursachen. Dann könnte sich wieder die Frage stellen, ob bei unvermeidbarer Priorisierung auch der Impfstatus berücksichtigt werden dürfte, wenn Betroffene durch Unterlassen einer empfohlenen und ihnen möglichen Impfung das Risiko ihrer jetzt eingetretenen, pandemiebedingten lebensbedrohlichen Erkrankung stark erhöht hatten.

Die Empfehlungen medizinischer Fachgesellschaften in Deutschland und der Schweiz zum Umgang mit der COVID-19-Pandemie gehen davon aus, dass der Impfstatus *nicht* als Differenzierungskriterium herangezogen werden dürfe (DIVI et al. 2021, S. 5; SAMW et al. 2021, S. 3, 5).^1^ Diese Empfehlungen sehen zwei Maßnahmen zur Bewältigung extremer Notlagen vor. Zunächst sollen, wenn sich eine Überlastungssituation abzeichnet, Behandlungen mit geringerer Dringlichkeit aufgeschoben werden (DIVI et al. 2021, S. 4; SAMW et al. 2021, S. 2). Für den Fall einer trotzdem eintretenden Ressourcenknappheit, die sich nicht mehr organisatorisch bewältigen lässt, etwa durch die Verlegung von Patienten, empfehlen sie Verfahren zur Entscheidungsfindung (Mehraugen-Prinzip) sowie das Kriterium der klinischen Erfolgsaussicht. Die Priorisierungsentscheidungen müssen sich dabei auf *alle* Patienten (nicht nur die an COVID-19 Erkrankten) beziehen, die intensivmedizinische Behandlung benötigen (DIVI et al. 2021, S. 5; SAMW et al. 2021, S. 3). Es komme darauf an, wie bei zu vergleichenden Patienten jeweils die Wahrscheinlichkeit sei, dass sie den aktuellen Zustand durch Intensivtherapie überleben (DIVI et al. 2021, S. 5).^2^ Die Empfehlungen für die Schweiz geben ebenfalls vor, dass „die kurzfristige Überlebensprognose das erste und wichtigste Entscheidungskriterium“ sein müsse (SAMW et al. 2021, S. 4).^3^ Das deutsche Bundesverfassungsgericht hat im Dezember 2021 eine gesetzliche Regelung für Situationen der Triage gefordert, aber keine verfassungsrechtlichen Bedenken gegen das Kriterium der klinischen Erfolgsaussicht geäußert (BVerfG, Beschluss vom 16.12.2021, 1 BvR 1541/20, Rdn. 116, 118). Der gegenwärtig (August 2022) diskutierte Entwurf eines neuen § 5c IfSG stellt ebenfalls auf die aktuelle und kurzfristige Überlebenswahrscheinlichkeit ab.

Anliegen meines Beitrags ist nicht, das Priorisierungskriterium der klinischen Erfolgsaussicht bzw. kurzfristigen Überlebensprognose zu kritisieren. Im Fokus steht vielmehr nur ein nachgeordnetes, im Anwendungsbereich enges Szenario: wenn eine Auswahl zwischen Patienten unvermeidbar würde, für die das Kriterium „kurzfristige Überlebensprognose“ keinen Unterschied ergibt. Wäre es dann, und nur dann, in Abweichung von den geschilderten fachgesellschaftlichen Empfehlungen *doch vertretbar*, ggf. Unterschiede im Impfstatus zu berücksichtigen, soweit Behandlungsbedürftigkeit durch die pandemisch verbreitete Erkrankung verursacht wurde? Oder ist darauf zu insistieren, dass es auch in Extremsituationen einer massiven Überlastung *niemals* eine Rolle spielen darf, ob Patienten geimpft oder ungeimpft sind? Oder sollte womöglich sogar noch stärker formuliert werden, dass der Impfstatus berücksichtigt werden *sollte, *eventuell sogar als vorrangiges Kriterium? Der erwähnte Gesetzesentwurf enthält keine Festlegungen zur Relevanz des Impfstatus.

Im Folgenden werden die möglichen Positionen und Argumente erörtert, ohne alle Fragen der Triage (oder gar noch umfassender: der Allokation knapper Ressourcen im Gesundheitswesen) anzusprechen. Im Ergebnis werde ich (gegen die erwähnten Empfehlungen der medizinischen Fachgesellschaften) die Position verteidigen, dass es vertretbar ist, unter bestimmten Umständen auch den Impfstatus zu berücksichtigen.

## These 1: Debatten zum Thema „Impfstatus“ sind unnötig

Die einfachste Begründung dafür, eine emotional wie intellektuell schwierige Debatte nicht zu führen, wäre, dass diese aus tatsächlichen Gründen überflüssig sei. Diese Schlussfolgerung könnte in der gegenwärtigen Lage (August 2022) schon deshalb naheliegen, weil unklar ist, ob im kommenden Winter mit einer schweren Pandemiewelle zu rechnen ist. Unausgesprochen könnte hinter dem Verdikt der medizinischen Fachgesellschaften gegen das Kriterium „Impfstatus“ *außerdem auch* die Hoffnung stehen, dass mit dem Primärkriterium „klinische Erfolgsaussicht/kurzfristige Überlebensprognose“ alle schwierigen Entscheidungen zu bewältigen seien und darauf verzichtet werden könne, über weitere Kriterien nachzudenken. Aber könnten in möglichen zukünftigen Pandemien wirklich zuverlässig *alle* unvermeidbaren harten Entscheidungen mit dem Auswahlkriterium „klinische Erfolgsaussicht/kurzfristige Überlebensprognose“ getroffen werden? Dies würde voraussetzen, dass ein Vergleich von zwei (oder mehr) Patienten ausnahmslos Abstufungen ergibt, was die Wahrscheinlichkeit des kurzfristigen Überlebens bei intensivmedizinischer Behandlung betrifft. Solange eine akute Überlastung nur punktuell und selten vorkommen würde, ist gut vorstellbar, dass bei jedem der unvermeidbaren Vergleiche von Überlebenswahrscheinlichkeit Unterschiede zu finden sind und eine Pandemielage durchstanden wird, ohne zusätzliche Auswahlkriterien heranzuziehen und ohne auf Zufallsentscheide wie Losen zurückzufallen. Im Review-Verfahren, das mein Diskussionsbeitrag durchlaufen hat, kam ein Gutachter zu der Einschätzung, dass die Situation der nicht ausreichenden Differenzierungsmöglichkeit „extrem unwahrscheinlich“ sei. Aber es ist nicht kategorisch auszuschließen, dass es Szenarien geben könnte, in denen die Anwendung von Prognosekriterien keinen erkennbaren Unterschied ergibt (Bhatt et al. 2021; Bormann 2021, S. 119; Walter 2020, S. 663). Falls die Zahl der behandelten und potenziellen Patienten, die intensivmedizinisch behandelt werden müssen, stark und schnell wächst, werden zahlreiche Priorisierungsentscheidungen erforderlich. Je mehr Entscheidungen insgesamt zu treffen sind, umso öfter wäre damit zu rechnen, dass unter den konkret betroffenen Patienten auch solche sind, bei denen keine Unterschiede bei der kurzfristigen Überlebensprognose auszumachen sind. Auch dann, wenn bei einzelnen klinischen Werten minimale Unterschiede bestehen, schwindet die normative Tragkraft (Sowada 2021, S. 304). Ein günstigerer Laborwert oder Score wird normativ nur dann relevant, wenn damit das klare Urteil verbunden werden kann, dass deshalb auch die Überlebensprognose besser ausfällt. Ansonsten ergeben numerisch ausdrückbare Werte allenfalls ein formales Entscheidungskriterium, ohne dass wirklich relevante Unterschiede bei der klinischen Erfolgsaussicht bestehen. In solchen Fällen würde der Sache nach ein Zufallsentscheid vorliegen, der jedoch nicht offengelegt würde. Insgesamt wird man nicht zuversichtlich sagen können, dass es vollkommen überflüssig sei, auch nur darüber nachzudenken, welche Auswahlkriterien in einem Entscheidungsbaum bei sehr ähnlicher kurzfristiger Überlebensprognose herangezogen werden könnten.

## These 2: Debatten zum Thema „Impfstatus“ sollten unterbleiben, weil sie zur Spaltung der Gesellschaft beitragen

Zunächst ist allerdings kurz zu erörtern, ob das Thema „Impfstatus“ ausgeblendet werden sollte, nicht weil ein solches Auswahlkriterium überflüssig wäre, sondern weil entsprechende Debatten heikel sind. Der Vorschlag, medizinische Versorgung vom Impfstatus abhängig zu machen, stößt bei vielen auf starke Ablehnung. Aus eigener Erfahrung kann ich nach einer ersten knappen Stellungnahme pro Berücksichtigung des Impfstatus im Kontext der COVID-19-Pandemie (Hörnle 2021) von entsprechend negativen Reaktionen berichten. Die Zahl war zu gering für eine ernsthafte soziologische Auswertung, und Leserzuschriften sind nicht repräsentativ für die Gesamtbevölkerung. Aber es ergab sich der erste Eindruck, dass die negative emotionale Reaktion zwei unterschiedliche Gruppen eint: zum einen überzeugte Impfgegner, die solche Vorstöße als persönliche Abwertung und Abstrafung wahrnehmen; zum anderen Personen, die betonen, selbst geimpft zu sein, aber als beunruhigend empfinden, was sie als Beitrag zur weiteren Entsolidarisierung und Kälte in modernen Gesellschaften wahrnehmen. Bereits von der Diskussion entsprechender Vorschläge wird vor allem in stark fragmentierten Gesellschaften wie in den USA ein Verlust an Vertrauen in Institutionen der Gesundheitspflege befürchtet (Chuan et al. 2021; Schuman et al. 2022, S. 2 f.).

Auf normative Begründungen für die Position „Impfstatus *darf nie* berücksichtigt werden“ ist noch einzugehen (These 3). Unabhängig davon ist zu erwägen, ob nicht schon der Umstand, dass negative Emotionen und Polarisierung zu erwarten sind, Grund sein könnte, Themen nicht öffentlich zu erörtern. Teilweise hängt die Antwort vom Diskussionskontext ab. In politischen Entscheidungsprozessen, die auf Kompromisse angewiesen sind, mag es nachvollziehbar sein, dass strittige Punkte ausgeklammert oder nicht intensiv erörtert werden, vor allem dann, wenn ein Kompromiss schnell gefunden werden muss und in den allermeisten Fällen auch ohne ein besonders umstrittenes Kriterium auszukommen wäre. Aus der Logik des Systems „Politik“ ergibt sich aber nicht, dass auch schon auf der Ebene von öffentlichen Debatten und wissenschaftlichem, d. h. medizinethischem oder rechtsphilosophischem Austausch emotionale und sozialpsychologische Gründe den Ausschlag geben müssten. Vor allem für spezialisierte, wissenschaftsnahe Debatten würde ich darauf bestehen, dass Argumente zur Rolle des Impfstatus nicht schon wegen des Verweises auf das damit verbundene Konfliktpotential außer Acht gelassen werden sollten.

## These 3: Der Impfstatus darf niemals berücksichtigt werden

Die These, dass der Impfstatus nicht berücksichtigt werden dürfe, kann auf zwei unterschiedlichen Wegen begründet werden: mit Rekurs auf individuelle Rechte oder mit Verweis auf kollektive Interessen an einem Gesundheitssystem, in dem das Vorverhalten von Patienten keine Rolle spielt. Die klinisch-ethischen Empfehlungen verweisen nur sehr knapp auf das „Gleichheitsgebot“ (DIVI et al. 2021, S. 5), um zu erläutern, warum der SARS-CoV-2-Impfstatus keine Grundlage für Priorisierung sein dürfe. Es wird nicht klar, ob damit ein individuelles Recht auf Gleichbehandlung gemeint ist oder eine gleichheitsorientierte Gesundheitsversorgung. Die Empfehlungen für die Schweiz gehen davon aus, dass der „Respekt vor dem Wert des Lebens jeder und jedes Einzelnen“ verlange, dass nicht auf Entscheidungen oder Handlungen des Einzelnen abgestellt werden dürfe. Solche sehr generellen Bezugnahmen auf Gleichheit oder Respekt bedürfen einer näheren Begründung.

### Menschenrechte und Menschenwürde

Kritiker einer Berücksichtigung des Impfstatus verweisen auf Menschenrechte und die Menschenwürde. So argumentiert etwa Beate Rudolf, die Direktorin des Deutschen Instituts für Menschenrechte, in einer Erklärung vom Dezember 2021.^4^ Diese Position hat allerdings viel weitreichendere Folgen als nur die Ablehnung des Impfstatus als ein nachrangiges Auswahlkriterium. Die dahinter stehende Logik versteht jede Benachteiligung eines Patienten auf der Basis eines personenbezogenen Sachkriteriums als Urteil, dass dieses Leben weniger wert sei. Selbst das Abstellen auf geringere Erfolgsaussicht der intensivmedizinischen Behandlung soll unzulässig sein, denn „ein absehbarer Tod oder eine kurze Lebensdauer“ seien „kein Grund, einen Menschen zugunsten eines anderen zu opfern“ (Deutsches Institut für Menschenrechte 2021, S. 69). Wenn Menschen nicht entscheiden sollen, muss zwangsläufig die Auswahl, wer die intensivmedizinischen Ressourcen bekommt, dem Zufall überlassen bleiben. Es blieben nur zeitliche Priorität (wer zuerst eingetroffen ist) sowie weitere Zufallsverfahren wie Losen (Deutsches Institut für Menschenrechte 2021, 2020; für Zufallsverfahren auch Sternberg-Lieben 2020, S. 634; Walter 2020, S. 667 ff.; Engländer 2021, S. 139 ff.; Fateh-Moghadam und Gutmann 2021, S. 306 f.; Zimmermann 2021, S. 249 ff.).^5^

Dieser Ansatz stößt auf Einwände. Der Verweis auf Zufall als einziges Auswahlkriterium bedeutet für diejenigen eine Zumutung, die in einer Pandemie die Hauptlast zu tragen haben, nämlich die Behandelnden in Krankenhäusern. Sie sollen die vertrauten Parameter klinischen Handelns und alle professionsethischen Muster über Bord werfen – und was tun? Die Vorschläge, auf den Zufall zu setzen, werden von den Befürwortern nicht näher spezifiziert. Soll gewürfelt oder sollen Streichhölzer gezogen werden, müsste ein TÜV-zertifiziertes Zufallsentscheidungsgerät angeschafft werden? Solche Vorstellungen stoßen nicht nur bei Ärzten und Pflegekräften auf Ablehnung (s. z. B. Marckmann et al. 2020, S. 176; SAMW et al. 2021, S. 5), sondern es wird beim Nachdenken über die Details auch klar, dass das hehre Ziel nicht zu erreichen ist, wie in Normallagen unbedingt alle zentralen Grundrechte und die daraus resultierenden Schutzansprüche zu respektieren. Auch mit Zufallsverfahren ist notwendigerweise ein veränderter Blick auf grundlegende Individualrechte verbunden. Wer den Zufallsgenerator oder Würfelbecher hervorholt, trifft damit folgende Aussage gegenüber jedem der betroffenen Patienten: Dein Recht auf Leben wird nicht mehr als volles Recht anerkannt, sondern nur noch als eine 50-prozentige, 33-prozentige, 25-prozentige etc. Chance auf Weiterleben. Dass dies gegenüber den Betroffenen würdiger oder respektvoller sein soll als ein Vergleich der diagnostisch begründeten Überlebenswahrscheinlichkeit oder des Impfstatus, versteht sich nicht von selbst (Birnbacher 2021, S. 213; krit. gegenüber Zufallsverfahren auch Aebi-Müller 2021, S. 23 f.; Kubiciel 2021, S. 226 f.).

Zur Untermauerung der These, dass es unzulässig sei, die klinische Erfolgsaussicht zu berücksichtigen, wird auf einen Satz in der Entscheidung des Bundesverfassungsgerichts zum Luftsicherheitsgesetz verwiesen, der lautet „Menschliches Leben und menschliche Würde genießen ohne Rücksicht auf die Dauer der physischen Existenz des einzelnen Menschen gleichen verfassungsrechtlichen Schutz“ (BVerfGE 115, 118, 158, Rdn. 132). Das klingt jedoch nur auf den ersten Blick einschlägig für Priorisierung in der Intensivmedizin. Verkannt wird, dass der Kontext in mehrfacher Hinsicht anders war. Würde die Luftwaffe in einem 9/11-Szenario ein entführtes Passagierflugzeug abschießen, um wenigstens die Menschen am Ort des von den Entführern angestrebten Aufschlags zu retten, würden die Passagiere durch aktives Tun getötet. Eine aktive Tötung macht eine andere Prüfungsstruktur erforderlich als die Entscheidung zwischen symmetrischen, gegenüber Patienten gleichermaßen bestehenden Rettungspflichten (Lübbe 2021b, S. 267 Fn. 10; Taupitz 2020, S. 442 f.).^6^

Der Verweis auf Menschenrechte ist nur dann nachvollziehbar, wenn Auswahlkriterien in Betracht gezogen würden, die mit Art. 3 Grundgesetz unvereinbar wären, z. B. sozialer Status. Im Übrigen führt der Rekurs auf Menschenrechte und Menschenwürde nicht weiter, insbesondere lässt sich damit nicht der schreckliche Umstand überspielen, dass jede Entscheidung, ob zufallsgeneriert oder begründet, dazu führt, dass ein Mensch sterben wird (Volkmann 2021). Im konkreten Kontext einer pandemiebedingten Notlage in Intensivstationen ist mit einer Priorisierungsentscheidung nicht die Aussage verknüpft, dass die ausgewählt Person „wertvoller“ und die nicht ausgewählte Person „weniger wert“ sei (Birnbacher 2021, S. 213; Lübbe 2021b, S. 274 ff.; Poscher 2021, S. 77). Weil verfassungsrechtliche Verweise auf Grundrechte und insbesondere auf die Menschenwürde nur in sehr beschränktem Maß weiterführen, liegt meinen Überlegungen keine spezifisch juristische Herangehensweise zugrunde. Aus verfassungsrechtlicher Perspektive ist lediglich zu konstatieren, dass eine Abwägung von Leben gegen Leben nicht prinzipiell ausgeschlossen ist und dass das Grundgesetz keine prinzipielle Schranke gegen „gut begründete und weithin geteilte ethische Vorstellungen“ als Leitlinie für Priorisierung errichtet (Poscher 2021, S. 79).

### Konsistenz- und Dammbruchargumente

Bedenken gegen die Berücksichtigung des Impfstatus werden vor allem darauf gestützt, dass ein solches Auswahlkriterium einen Bruch mit üblichen Grundsätzen der medizinischen Versorgung bedeute. In der Pressemeldung zur neuesten Fassung der klinisch-ethischen Empfehlungen verweist Georg Marckmann darauf, dass bei lebensbedrohlichen Erkrankungen Hilfspflichten grundsätzlich unabhängig vom Vorverhalten bestehen.^7^ Eine entscheidende Frage ist, ob es ein Konsistenzgebot gibt, das ausschließt, auch in den extremsten Lagen einer Pandemie in der Intensivmedizin das Vorverhalten von Patienten zu berücksichtigen. Dies würde ich verneinen. Selbstverständlich ist vom *Grundsatz* einer unbedingten intensivmedizinischen Versorgungspflicht auszugehen, und von der damit verbundenen Verpflichtung, eine bedarfsangemessene, Sicherheitsreserven einschließende Zahl an intensivmedizinischen Behandlungsplätzen vorzuhalten. Wenn aber in Extremlagen einer Pandemie Personal und Ressourcen nicht mehr ausreichen, sind so ungewöhnliche Entscheidungen erforderlich, dass es kein absolutes Konsistenzgebot geben kann.

Während sich Konsistenzargumente auf die bislang angewandten Entscheidungsregeln beziehen, weisen Dammbruch- oder *slippery slope*-Argumente in die Zukunft. Wäre es ein Argument gegen die Berücksichtigung des Impfstatus, dass dies das Gesundheitssystem der Zukunft in vielen Bereichen, auch jenseits der Intensivmedizin, verändern könne? Führt eine „schiefe Ebene“ von der Berücksichtigung des Impfstatus im Kontext einer Pandemie dazu, dass bald auch in Normallagen wegen gesundheitsschädlicher Lebensführung (Rauchen, Alkoholkonsum, Übergewicht) oder riskanten Aktivitäten (manche Sportarten, schnelles Fahren) Behandlungen versagt werden? Zwei Gesichtspunkte sind zu unterscheiden. Die Befürchtung einer schiefen Ebene oder eines Dammbruchs würde voraussetzen, dass der Ausbau der vereinzelt schon gesetzlich vorgesehenen Berücksichtigung von Eigenverursachung (Huster 2022) negativ zu bewerten wäre. Dies kann und muss hier nicht erörtert werden. Soweit es um die Organisation des Gesundheitssystems in Normallagen geht, gibt es überzeugende pragmatische wie normative Gründe, die gegen eine umfassende, konsequente Berücksichtigung von Vorverhalten sprechen (Probleme bei der Sachverhaltsaufklärung; Präferenz für Solidarität, die auch menschliche Schwächen toleriert und finanziell mitträgt). Unser Thema betrifft sehr extreme Situationen, in denen infolge einer Pandemie der Tod von Menschen zu erwarten wäre, die wegen extremster Überlastung von Krankenhäusern nicht mehr behandelt werden könnten. In solchen Extremlagen ist es unvermeidbar, dass es zu Brüchen kommt und Konsistenzgebote nicht mehr handlungsleitend sein können. Vorschläge für Ausnahmesituationen determinieren nicht zwangsläufig oder mit hoher Wahrscheinlichkeit zukünftige normative Debatten zur Organisation des Gesundheitswesens (Huster 2021, S. 92 ff.).

## These 4: Der Impfstatus sollte berücksichtigt werden, um die Impfquote zu erhöhen

Die mögliche Rolle des Impfstatus als Entscheidungskriterium ist nicht schon dann geklärt, wenn es gelingt, Einwände zu den „kategorisch ausgeschlossen“-Argumenten zu präsentieren. Es bleiben dann immer noch zwei zu diskutierende Alternativen. Zum einen könnte in Empfehlungen festgehalten werden, dass auch auf den Impfstatus abgestellt werden *darf*, soweit dies im Einzelfall zur Auflösung eines Entscheidungsdilemmas erforderlich ist (s. dazu These 6). Zum anderen ist eine weitergehende Forderung zu bedenken. Diese würde lauten, dass der Impfstatus in vielen Fällen als ausschlaggebend berücksichtigen werden *soll*, etwa immer dann, sobald ein gewisser Schwellenwert bei der klinischen Erfolgsaussicht überschritten ist. In diese Richtung weisen Forderungen aus der Verhaltensökonomie.^8^

Der Zeitpunkt, der aus dieser Perspektive in den Vordergrund rückt, ist nicht der einer verzweifelten Entscheidungslage in der Notfall- und Intensivmedizin, sondern die vorlagerte Zeit, in der sich Menschen für oder gegen eine Impfung entscheiden. Das verfolgte Ziel ist ein kollektives: die Erreichung einer möglichst hohen Impfquote. Die Information, dass Ungeimpfte im Falle einer lebensbedrohlichen Erkrankung bei unvermeidlicher Priorisierung zurücktreten müssten, könnte ein Mittel zur Verbesserung der Impfquote sein. Zwingender Bestandteil einer solchen Strategie wäre es, diese mögliche Folge in der Öffentlichkeit nachdrücklich zu betonen.

Es lohnt sich, eine solche Option ernsthaft zu durchdenken. Sollte sie sich als vielversprechend erweisen, könnte möglicherweise auf eine Impfpflicht verzichtet werden (Hoffmann 2021). Die mit einer gesetzlichen Impfpflicht verbundene normative Begründungslast sowie die praktischen Herausforderungen würden entfallen. Es ist absehbar, dass die Aufforderung, sich impfen zu lassen, gegenüber Personen, die sich faktisch entziehen oder sich rechtlich zur Wehr setzen, nicht oder nicht zeitnah durchzusetzen ist. Das in politischen Debatten zu hörende Argument, dass sich erfahrungsgemäß viele Menschen auch ohne Vollstreckungsdruck rechtskonform verhalten und deshalb eine gesetzliche Impfpflicht auch mit bruchstückhafter Kontrolle und ohne spürbare Sanktionen verhaltenssteuernd wirken würde, überzeugt nicht – die konformitätsorientierte Mehrheit ist im Zweifel bereits geimpft.

Bei einer konsequentialistischen, auf die Erhöhung der Impfquote zielenden Begründung von Priorisierungskriterien ist die Hauptfrage, ob die Ankündigung „Nachrang bei Priorisierung“ besser geeignet wäre, Personen zu motivieren, die bislang nicht impfwillig waren. Tendenziell dagegen sprechen allgemeine verhaltensökonomische Erkenntnisse: Menschen richten ihr Verhalten auch in medizinischen Kontexten nicht in geradlinig-rationaler Weise an sachlichen Informationen und Risikobewertungen aus. Vielmehr müssen alle Konzepte zur Erhöhung der Impfquote berücksichtigen, dass es komplexe Interaktionen mit anderen psychologischen Wirkmechanismen gibt (Soofi et al. 2020). Die Wirkung von positiven Anreizen (etwa bescheidene finanzielle Vorteile) auf die Impfquote scheint gering zu sein oder sogar gegenteilige Effekte zu erzeugen (Chang et al. 2021; Robertson et al. 2021). Offen ist, ob starke negative Aussagen wie die Androhung, ggf. nicht intensivmedizinisch versorgt zu werden, Effekte hätten. Angesichts der hochgradig emotionalen Aufladung und irrationaler Züge bei Impfgegnern ist es eher unwahrscheinlich, dass Impfgegner und passiv Verharrende dadurch in relevanter Zahl zu beeinflussen wären.

## These 5: Der Impfstatus sollte immer berücksichtigt werden, weil dies eine gerechte Antwort auf vorwerfbares bzw. verdienstvolles Verhalten ist

Auf Nachweisprobleme in Sachen effektive Verhaltenssteuerung käme es nicht an, wenn der Impfstatus schon deshalb zu berücksichtigen wäre, weil es sich um eine genuin gerechte Lösung handele. Es ist auf den ersten Blick nicht ganz fernliegend, Überlegungen zu Verdienst und Tadel anzustellen. Sich impfen zu lassen, dient nicht nur persönlichen Schutzinteressen, sondern auch dem Gemeinwohl und ist deshalb lobenswert. Wer sich dafür entscheidet, nimmt Unannehmlichkeiten und Risiken in Kauf: Zeitaufwand für die Organisation und das Aufsuchen einer Praxis oder eines Impfzentrums; die nicht selten auftretenden Beschwerden im unmittelbaren Anschluss an die Impfung; das Risiko von erheblichen Gesundheitsfolgen (ein sehr kleines, aber bestehendes Risiko, s. Paul-Ehrlich-Institut 2021; Myles et al. 2021). Gleichzeitig trägt jede einzelne Impfung dazu bei, dass das Problem nicht entsteht, das Priorisierung erst erforderlich macht, nämlich die Überlastung von Krankenhäusern. Wer sich dagegen nicht impfen lässt, erspart sich einerseits persönliche Risiken und Aufwand und trägt anderseits zur Existenz und Größe der Gruppe der Ungeimpften bei, die wiederum als Gruppe der maßgebliche Grund für überlastete Intensivstationen sind.

Der Gedanke, dass die ethische Qualität der Vorentscheidungen von Patienten vermessen werden könnte, führt jedoch aus mehreren Gründen nicht viel weiter. Erstens sind in der Notfall- und Intensivmedizin umfassende Gerechtigkeitsurteile schon aus praktischen Gründen nicht möglich. Es ist unmöglich, Fakten zu ermitteln, die für anspruchsvolle, einzelfallbezogene Gerechtigkeitsurteile relevant werden. Zum Beispiel ist vorstellbar, dass der genauere Blick auf die Lebensumstände enthüllen könnte, dass das Verhalten eines ungeimpften Patienten letztlich verdienstvoller war als das des geimpften konkurrierenden Patienten (Chuan et al. 2021), etwa wenn sich Ersterer extrem isoliert hatte, Letzterer aber trotz des Umstands, dass Virenübertragung möglich blieb, täglich Clubs aufsuchte. Zweitens gibt es grundsätzliche Bedenken gegen eine an Lob und Tadel ausgerichtete Herangehensweise. Als generelle Regel gilt: Das Gesundheitssystem ist nicht dafür zuständig, menschliches Verhalten umfassend ethisch zu bewerten (Schuman et al. 2022, S. 2) oder gar Strafen aufzuerlegen. Hinzu kommt ein weiteres Problem: die Frage der Proportionalität. Nach einer verdienstorientierten Logik sollten Bestrafung und Belohnung in Proportion zum bewerteten Individualverhalten stehen. Die Zuweisung lebensrettender intensivmedizinischer Ressourcen ist jedoch wegen ihres Gewichts als Entscheidung über Leben und Tod nicht passend als Untermauerung von Tadel und Verdienst für unterbliebene und vollzogene Impfung. Sie wäre als Strafe disproportional. Proportionalität (des Verdiensts) wird im Übrigen auch mit Blick auf erfolgte Impfung in Frage gestellt, weil die lebensrettenden Effekte einer individuellen Impfung minimal sind (Parker 2021).

## These 6: Der Impfstatus darf dann berücksichtigt werden, wenn es für Entscheidende keine bessere Begründung gibt

Die vorstehend formulierte Skepsis gegenüber genuinen Gerechtigkeitsurteilen als Basis von Priorisierung auf Intensivstationen in Pandemien scheint nahezulegen, dass damit jede Berücksichtigung des Impfstatus ausscheide. Trotzdem komme ich zu dem Vorschlag, dass der Impfstatus unter bestimmten, eng umgrenzten Umständen berücksichtigt werden darf, nämlich dann, wenn das Kriterium „klinische Erfolgsaussicht/kurzfristige Überlebensprognose“ keine Differenzierung erlaubt und der benachteiligte Patient wegen der Krankheit in die lebensbedrohliche Lage geraten ist, die durch eine nachdrücklich und öffentlich empfohlene Impfung mit großer Wahrscheinlichkeit verhindert worden wäre. Wenn die erste Bedingung greift (keine Differenzierung nach kurzfristiger Überlebensprognose möglich), ist es unabdingbar, auf ein weiteres Auswahlkriterium zurückzugreifen, und an ernsthaft zu diskutierenden Alternativen bleibt an dieser Stelle des Entscheidungsbaums wenig. Man wird die Suche nach akzeptablen Auswahlkriterien für Extremsituationen in der Regel als Suche nach dem kleinsten Übel beschreiben müssen, und dies gilt in besonderem Maße, wenn das vorrangig anzuwendende Kriterium, das die medizinischen Fachgesellschaften für eine solche Extremsituation empfehlen, nicht zu einem Ergebnis führt. Die Alternative zur Berücksichtigung des Impfstatus könnte dann nur sein, ohne weitere Zwischenüberlegung zu losen oder ein anderes Zufallsverfahren einzusetzen. Zufallsverfahren sind jedoch eine besonders unbefriedigende Option (oben These 3); insbesondere ist den Behandelnden in der Extremsituation einer Pandemie nicht zumutbar, etwas zu tun, was ihrer Berufsethik grundlegend widerspricht. Zufallsverfahren sollten nur dann in Erwägung gezogen werden, wenn es wirklich keinerlei andere Möglichkeit mehr gibt (ähnlich skeptisch gegenüber Losverfahren Waßmer 2021, S. 348; Weigend 2021, S. 379 f.). Solange möglich, ist Zufallsverfahren ein Auswahlkriterium vorzuziehen, das auf Sachgründe verweist, die auch mit dem beruflichen Ethos von Medizinern vereinbar sind.

Zugegebenermaßen sind wegen der Notwendigkeit, schnell und notgedrungen mit unzureichendem Wissen über die Vorgeschichte zu entscheiden, fein kalibrierte Urteile über persönliche Schuld der nicht geimpften Person nicht möglich, und auf die Probleme, die mit anspruchsvollen, tadels- und verdienstbasierten Gerechtigkeitsurteilen verbunden sind, wurde bereits hingewiesen. Solange es keine gesetzliche Impfpflicht gibt, kann man es auch als nicht völlig konsistent kritisieren, wenn der Staat die Impfung zwar dringend empfiehlt, diese aber eine Sache persönlicher Entscheidung bleibt, gleichzeitig allerdings davon unter Umständen Priorisierung abhängen soll (Lübbe 2021a). Der Vorschlag, das Unterlassen der Impfung als ein Kriterium bei schwierigsten Entscheidungen zuzulassen, kann aus durchaus nachvollziehbaren Gründen kritisch analysiert werden (s. dazu Lübbe 2021a). Aber was dann? In Triage-Szenarien ist weder Passivität noch genaueres Überprüfen der Hintergründe eine Option, anders etwa als bei der Frage, ob später die Kosten einer Behandlung vom Patienten zurückgefordert werden könnten. Es *muss* entschieden werden, und das *schnell*. Wer ein mögliches Auswahlkriterium aus moralphilosophischer Sicht verwirft, sollte dies nur tun, wenn stattdessen eine bessere Alternative benannt wird.

Das für mich entscheidende Argument ist, dass im Vergleich mit einer Zufallsentscheidung das Abstellen auf unterlassene Impfung ggf. die – relativ gesehen – bessere Alternative wäre. Eine Priorisierung nach Impfstatus kann immerhin begründet werden, und zwar damit, dass auf den eigenen Anteil eines Patienten an der eigenen Notlage verwiesen wird. Wer in der Vergangenheit die Vermeidung von Impfrisiken oder sonstigen Unannehmlichkeiten höher gewichtet hat als die Aussicht, durch Impfung die Wahrscheinlichkeit lebensgefährlicher Erkrankung zu senken, muss es im Gegenzug hinnehmen, wenn diese eigene Entscheidung in Ermangelung besserer Auswahlkriterien zum Kriterium bei Priorisierung wird (Hoffmann 2021). Umfragen deuten darauf hin, dass dies auch in der Bevölkerung von einer großen Mehrheit so gesehen wird (Sprengholz et al. 2021). Ob die Entscheidung gegen eine Impfung hinreichend faktenbasiert war, ob sie auf Leichtgläubigkeit oder sogar auf hochgradig irrationalen Verschwörungstheorien beruhte, muss nicht erörtert werden – es kommt auf das Ergebnis an, also auf das wissentliche Unterlassen einer Impfung, die möglich gewesen wäre. Selbst wenn passives Verhalten mit Bequemlichkeit oder habituellem Desinteresse an Gesundheitsvorsorge zu erklären war, genügt der eigene Anteil am Ausbruch der eigenen schweren Erkrankung, um zu begründen, dass danach nicht verlangt werden kann, den letzten freien Behandlungsplatz zu verlosen.^9^

## Schlussbemerkung

Es ist zu bedauern, dass in den Empfehlungen der medizinischen Fachgesellschaften in Deutschland und der Schweiz die Option kategorisch ausgeschlossen wurde, bei vergleichbarer klinischer Erfolgsaussicht auf den Impfstatus abzustellen, falls einer der Betroffenen den lebensbedrohlichen Krankheitszustand durch die empfohlene und verfügbare Impfung hätte vermeiden können. Auch wenn mangels Wissen über die persönlichen Hintergründe der Entscheidung gegen eine Impfung ein anspruchsvolles Schuldurteil meist nicht möglich ist, bestehen keine durchschlagenden Einwände dagegen, den Beitrag zur eigenen Erkrankung und zur Überlastung der Intensivstationen zu berücksichtigen. Bessere Alternativen gibt es nicht. Insbesondere sind weder Zufallsverfahren wie Losen noch das Abstellen auf Laborwerte ohne tatsächliche Relevanz für die Überlebenswahrscheinlichkeit bessere Auswahlkriterien. Dies schließt es selbstverständlich nicht aus, den Entscheidenden zuzugestehen, den Impfstatus auszublenden, wenn die Unterlagen nahelegen, dass die Betroffenen keine Wahl gehabt haben könnten, etwa weil aus medizinischen Gründen nicht geimpft wurde.

Praktisch umsetzbar wäre der Vorschlag, dass die Entscheidenden den Impfstatus berücksichtigen dürfen, allerdings wohl nur dann, wenn es ein Impfregister gäbe, in das auch eingetragen würde, falls aus medizinischen Gründen nicht geimpft werden konnte. Ohne eine den Behandelnden zugängliche Dokumentation entstünde das Problem, dass Betroffene in Knappheitssituationen angeben könnten, ihre Impfnachweise verloren zu haben, um nicht zugunsten von tatsächlich Geimpften zurücktreten zu müssen. Als zweite Vorbedingung sollte kommuniziert werden, dass Vorverhalten eine Rolle spielen kann – dies gilt nicht nur, wenn Verhaltenssteuerung angestrebt wird (These 4), sondern auch aus Gründen der Fairness, da die Frage der Eigenverursachung bislang für unser Thema keine Rolle gespielt hat (Huster 2022).^10^

## Footnotes

1 Nicht erörtert wird der Impfstatus als mögliches Entscheidungskriterium in Empfehlungen aus dem Jahr 2020, s. z. B. Deutscher Ethikrat (2020), Bioethikkommission Österreich (2020).

2 S. zum Problem, dass diese Vorgabe zur Frage führt, wie lange der Überlebenszeitraum sein muss (ein paar Tage, Wochen, ein Jahr?) Dufner (2021, S. 226 f.).

3 Außerdem geben sie vor, dass Dauer und Aufwand der intensivmedizinischen Behandlung eine Rolle spielen dürfe, SAMW et al. (2021, S. 5).

4 S. https://www.tagesschau.de/newsticker/liveblog-coronavirus-donnerstag-275.html, zugegriffen: 23. Juni 2022. Der schriftliche Bericht des Instituts „Entwicklung der Menschenrechtssituation in Deutschland. Juli 2020–Juni 2021“ enthält einen Abschnitt zur Triage, der aber den Aspekt des Impfstatus nicht aufgreift.

5 Dass auch das Kriterium der Dringlichkeit der Behandlung berücksichtigt werden dürfe, spielt für unser Thema keine Rolle, weil Ausgangspunkt der Überlegungen ist, dass die pandemiebedingte Knappheit die Auswahl unter Erkrankten erzwingt, die alle ohne intensivmedizinische Behandlung versterben.

6 Die klinisch-ethischen Empfehlungen enthalten allerdings auch eine effizienzorientierte Begründung: Es solle möglichst wenigen Patienten eine Behandlungschance vorenthalten werden (DIVI et al. 2021, S. 4 f.). Aber die negativ-utilitaristische Begründung ist nicht die einzig mögliche, und sie ist, anders als beim erwogenen Flugzeugabschuss, wo die Ratio allein in der Maximierung geretteter Leben liegen kann, nicht der Entscheidungslage immanent. Eine utilitaristische Abwägung müsste vor allem die Zeitspanne einbeziehen, während derer ein Patient den intensivmedizinischen Behandlungsplatz benötigt, was mit dem Kriterium der klinischen Erfolgsaussicht nicht deckungsgleich ist (Lübbe 2021b, S. 262 f.).

7 https://www.divi.de/presse/pressemeldungen/pm-intensiv-und-notfallmediziner-aktualisieren-klinisch-ethische-empfehlungen-zur-priorisierung-und-triage-bei-covid-19. Zugegriffen: 13. Juli 2022.

8 S. für diesen Ansatz Armin Falk in einem Interview in der FAZ vom 29.07.2021, https://www.faz.net/aktuell/wirtschaft/mehr-wirtschaft/impfpflicht-von-leopoldina-forscher-armin-falk-gefordert-17460074.html, zugegriffen: 22. Juni 2022; außerdem Hank (2022).

9 Vorstellbar wäre es ferner, dies in extrem angespannten Situationen einer Pandemie auch auf anderes riskantes Vorverhalten auszuweiten, das in einem klar beschreibbaren Kausalzusammenhang zu schwerwiegenden Verletzungen steht, etwa, wenn ggf. mit Verweis auf die angespannte Lage in Krankenhäusern von bestimmten Risikosportarten abgeraten wurde.

10 Huster schlägt außerdem vor, die unterlassene Impfung nur bei älteren Personen einzubeziehen, bei denen das Risiko einer schwerwiegenden Erkrankung am Sars-Cov-2-Virus deutlich höher ist. Das halte ich nicht für zwingend.
